# Supplementary figures and images for: Pseudomonas aeruginosa cleaves the decoding center of Caenorhabditis elegans ribosomes
Source: PLoS Biol. 2020 Dec 1;18(12):e3000969. doi: 10.1371/journal.pbio.3000969 (PMC7707567; doi:10.1371/journal.pbio.3000969)

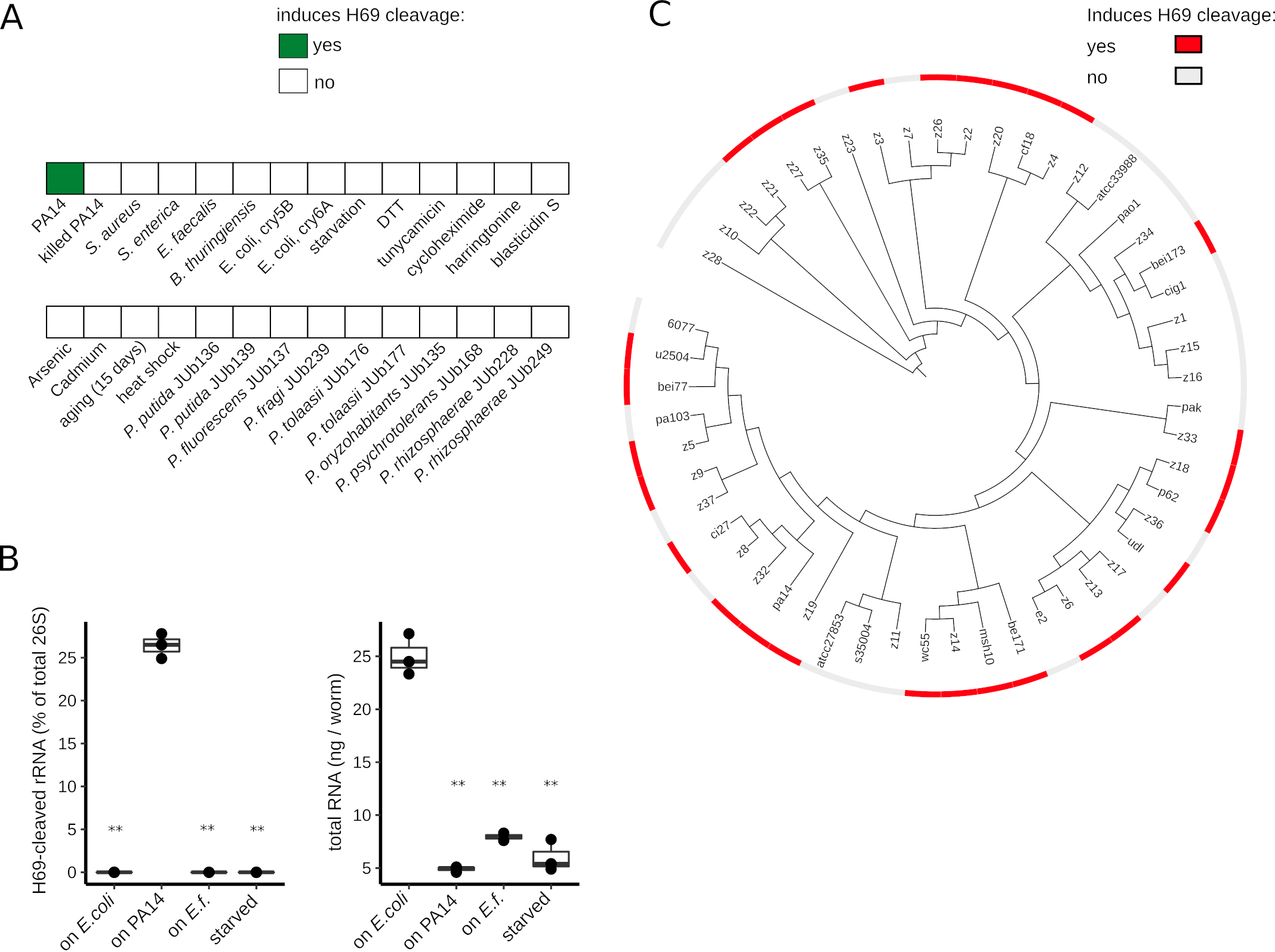

Supplement: S1 Fig — (A) Matrix summary of H69 cleavage induction by 28 different stresses and bacteria. Worms were exposed to the indicated condition for 24 h at 25°C, and worm total RNA was extracted and analyzed by capillary electrophoresis. Only PA14 induces cleavage at H69 (indicated by green box). (B) Matched measurement of H69 cleavage (left graph) and total RNA content (right graph) for wild-type worms exposed 24 h to either Pseudomonas aeruginosa PA14, Escherichia coli HB101, Enterococcus faecalis (E.f.), and starvation (starved). “**” indicates p-value < 0.01 for Welch t test comparison to P. ae. (left graph) or E. coli (right graph). S1 Data contains source data for panel B. (C) Collection of P. aeruginosa isolates tested for their capacity to induce H69 cleavage on worms (H69 cleavage–inducing capacity indicated by red color). The phylogenetic tree of the isolates was obtained from [27]. (TIF) [file pbio.3000969.s001.tif]

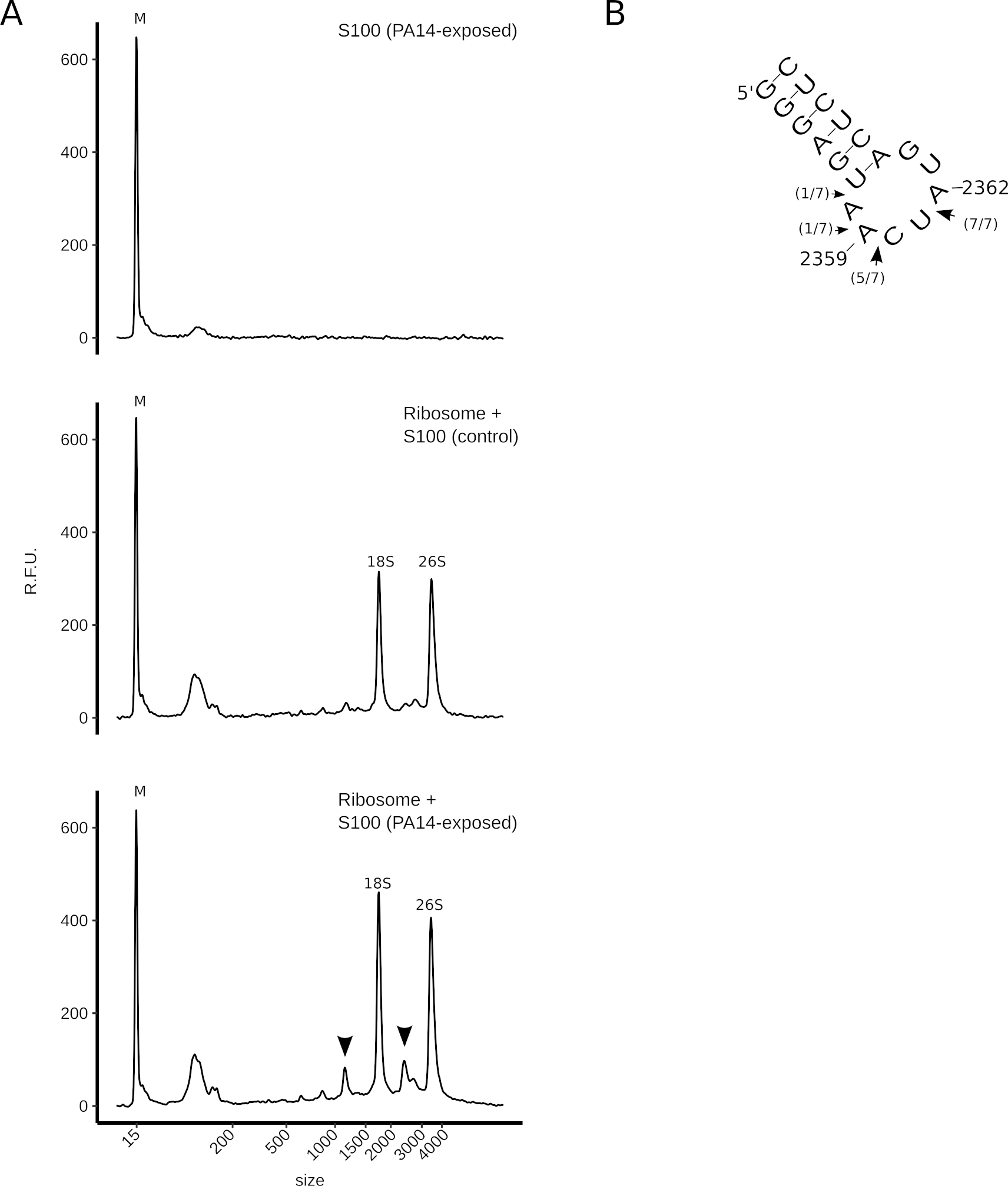

Supplement: S2 Fig — (A) RNA profile of the S100 extract from PA14-exposed worms, used for the in vitro assays (top panel); RNA profile of ribosomes after incubation with S100 extract from control (middle panel) or PA14-exposed worms (bottom panel). Arrowheads indicate bands at approximately 1,100 and approximately 2,300 nt. “M” indicates a 15-nt marker used in the electrophoretic separation system. (B) Termini of the 2 bands in (A) are indicated by arrowheads. The termini map to the H69 of 26S rRNA. The number of independent sequences mapped to the cut sites is shown in parenthesis. (TIF) [file pbio.3000969.s002.tif]

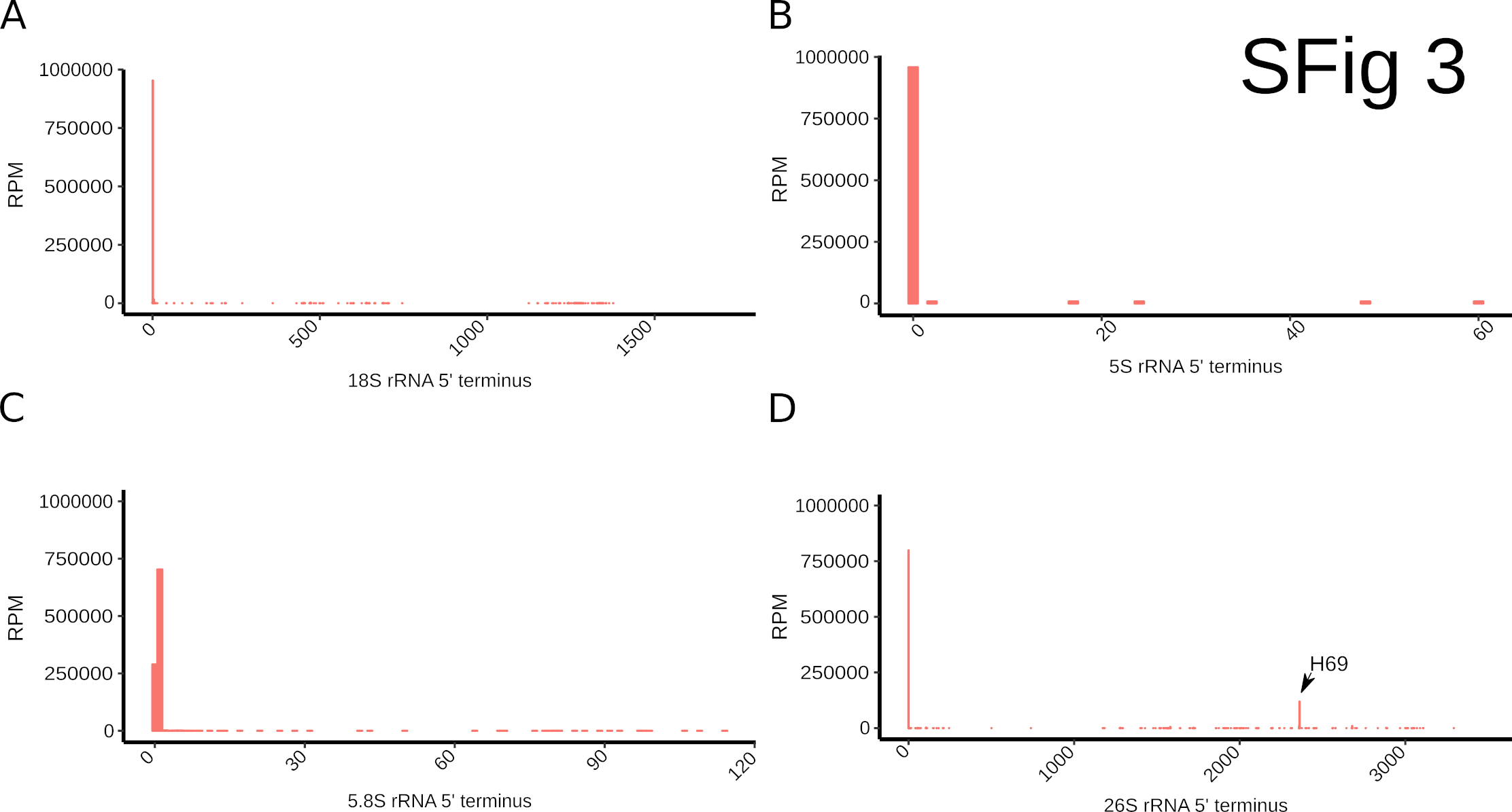

Supplement: S3 Fig — Frequency of 5′ termini along the worm 18S rRNA (A), 5S rRNA (B), 5.8S rRNA (C), and 26S rRNA (D) as determined by degradome sequencing. The H69 cleavage in the 26S rRNA is indicated by the black arrowhead. (TIF) [file pbio.3000969.s003.tif]

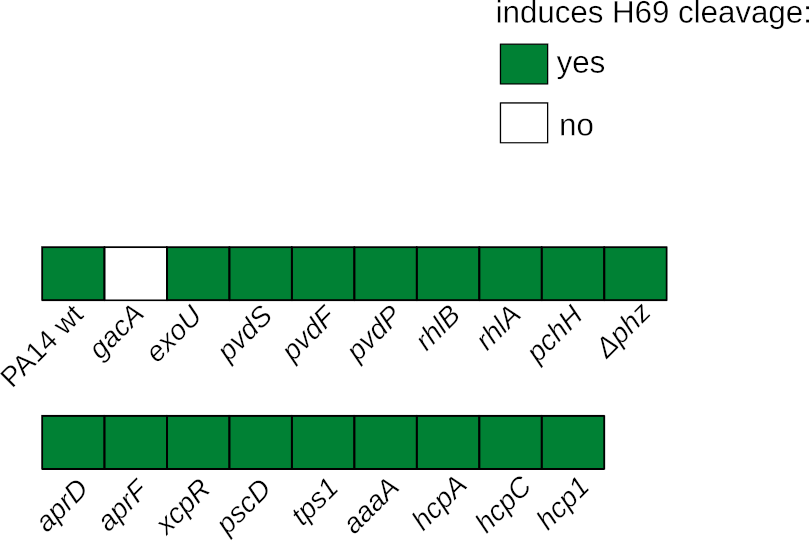

Supplement: S4 Fig — Matrix summary of H69 cleavage induction by a set of P. aeruginosa mutants in virulence genes (top row) and secretion systems (lower row). PA14 wt and gacA are included as controls. Worms were exposed to the indicated mutant bacteria for 24 h at 25°C, and worm total RNA was extracted and analyzed by capillary electrophoresis. Induction of H69 cleavage is indicated by the box color (green: cleavage level similar to PA14 wild type; white: absent cleavage). (TIF) [file pbio.3000969.s004.tif]

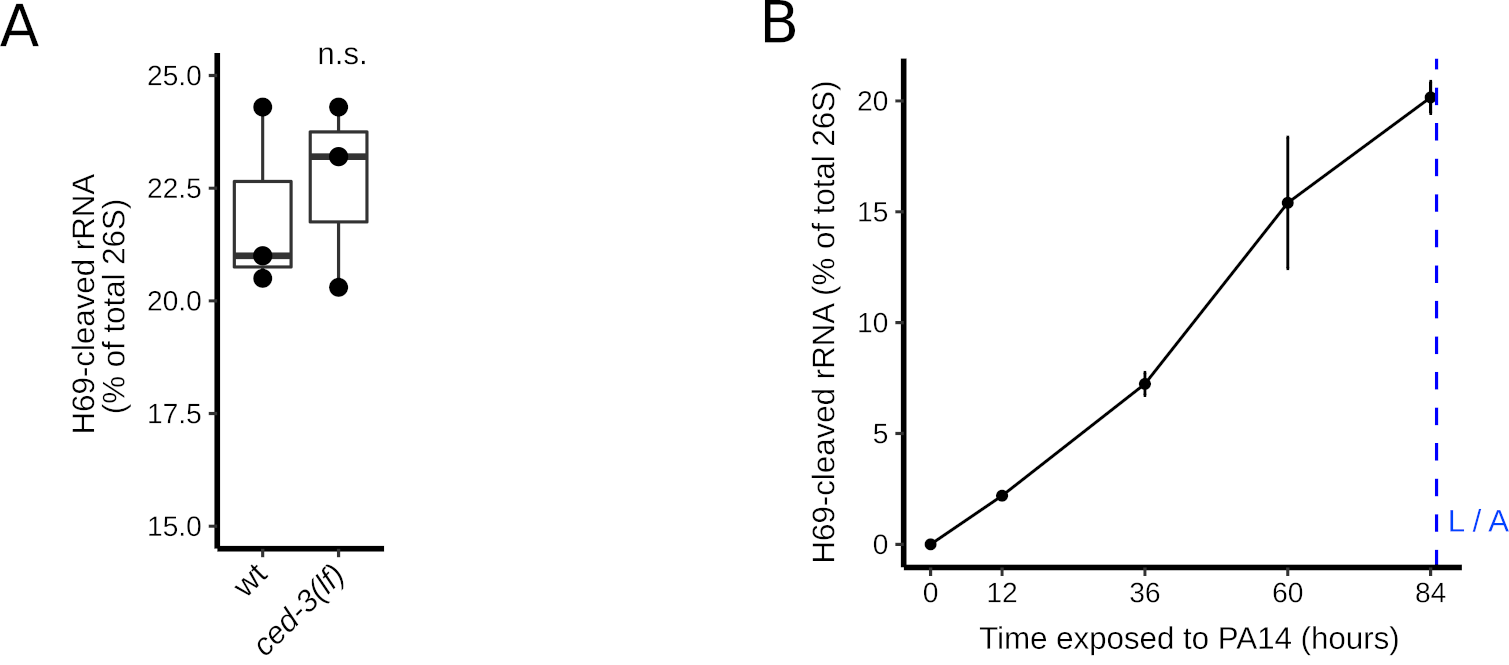

Supplement: S5 Fig — (A) H69 cleavage levels for a ced-3 loss of function (lf) mutant, which is defective in apoptosis. The cleavage levels are not significantly different (n.s.) than those of wild-type (wt) worms (Welch t test comparison, p-value > 0.05). (B) Graph of H69 cleavage levels in relationship to larval worm exposure time to PA14 (in hours). L1 larvae are experimentally set to start development on PA14 (t = 0) and transition from larvae to adult (L/A) at the time indicated by the dashed blue line (average time in hours). The average of tree measurements per timepoint and standard deviation (error bars) is plotted. S1 Data contains source data for all panels. (TIF) [file pbio.3000969.s005.tif]

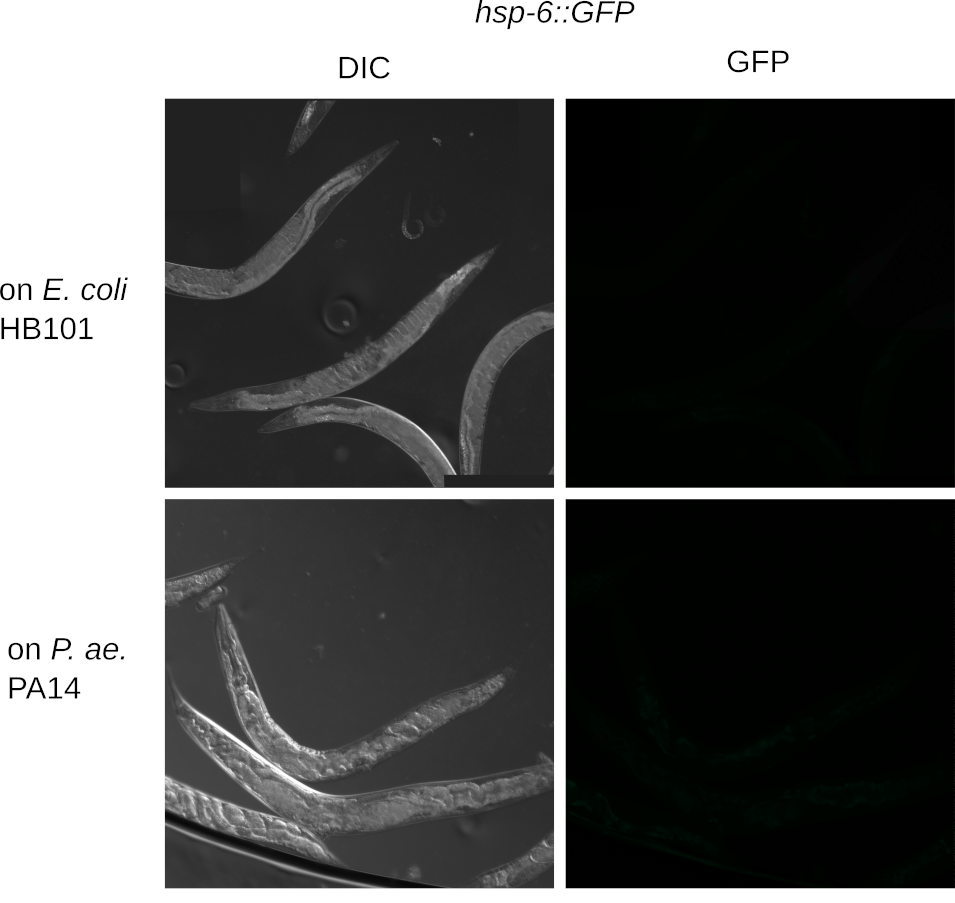

Supplement: S6 Fig — Fluorescent microscopy images of hsp-6::GFP worms exposed to PA14 or Escherichia coli HB101. Young adult worms of the indicated reporter were exposed to the respective bacteria for 24 h. GFP expression level is indicated by the green color. Nomarski images (DIC) corresponding to the fluorescent pictures are shown. (TIF) [file pbio.3000969.s006.tif]
