# Supplementary material for: Pseudomonas aeruginosa cleaves the decoding center of Caenorhabditis elegans ribosomes
Source: PLoS Biol. 2020 Dec 1;18(12):e3000969. doi: 10.1371/journal.pbio.3000969 (PMC7707567; doi:10.1371/journal.pbio.3000969)
Supplement: Attachment — Submitted filename: Vasquez-Rifo_Response_to_Reviewers.docx [file pbio.3000969.s012.docx]

**Detailed responses to reviewer report**

**Responses to Reviewer #1**

***Reviewer #1:***

*In "Pseudomonas aeruginosa cleaves the decoding center of Caenorhabditis elegans ribosomes," the authors show that the pathogen P. aeruginosa inhibits translation in the host (C. elegans) in part by a very specific cleavage (H69) of the 26S ribosomal RNA. Previous work had shown inhibition of translation by P. aeruginosa in a general manner via the action of Exotoxin A (ToxA). However, this new finding is novel and occurs independently of ToxA. While the exact bacterial factors involved as well as the mechanism are not identified, the authors show that the activity is dependent on important virulence/quorum sensing regulators such as GacA. They also shown that the process is dependent on host dynamin, possibly because the bacterial factor(s) involved are brought in through the endocytosis pathway. Finally, the authors demonstrate that three major host defense pathways, mediated by PMK-1, FSHR-1 and ZIP-2 antagonize this cleavage. The technical aspects of the manuscript are*

*very strong, and the story is clear and compelling. Identification of this new way in which P. aeruginosa manipulates host physiology is an important finding. However, the manuscript would be strengthened by further insight into the identity of the nuclease and whether this happens in hosts other than C. elegans.*

**Response**: We are thankful for the reviewer’s acknowledgment of the manuscript as technically robust, compelling, and describing an important finding. Our revisited manuscript and the responses below address the reviewer’s concerns.

*Major Comments:*

*1) Identification of the nuclease and/or more of the mechanism would really make this story.*

**Response**:

We share the reviewer's interest in the identity of the nuclease involved in the ribosome cleavage at H69. We have dedicated extensive and ongoing efforts to identify the H69 nuclease. However, Substantial work is still required before we can report the identification of the H69 nuclease. Genetic evaluation of candidate nucleases both in *P. aeruginosa* (using the PA14 transposon library) and *C. elegans* (testing of mutants when available, and/or gene knockdowns by RNAi) have led to no definitive identification of a H69 nuclease. However, our genetic screens of potential nucleases encoded in the *C. elegans* and *P. aeruginosa* genomes are not yet comprehensive.

In parallel with the genetically-driven approaches, we are also conducting a parallel approach to identify the nuclease through activity-based fractionation of lysates of infected worms, followed by enzymological and in vivo genetic validation. It is apparent that this effort will constitute a extensive body of experimental work whose timeframe for completion is still far from certain. Moreover, while we acknowledge that the identification of the nuclease would enhance the manuscript's narrative, we do not think that any of the presented claims or conclusions critically depends on such identification.

*2) Is there any evidence that this fascinating mechanism of translational inhibition occurs in mammalian systems? For example, have you looked for this cleavage event after infecting lung epithelium cells with PA14?*

**Response**:

We anticipate that the mechanism is likely to be conserved. H69 is a ribosomal structure of identical sequence in eukaryotes. Indeed, in preliminary studies and we have observed that upon infection of lung cells in cell culture, *P. aeruginosa* induces the accumulation of cleaved 28S ribosomal rRNA, similarly as observed with *C. elegans*. However, these studies are likewise in early stages, and substantial work remains before we could feel comfortable with reporting any definitive conclusions.

*3) Is the dynamin mutant/RNAi less sensitive to killing by P. aeuruginosa, as would be predicted?*

**Response**:

It is reasonable to predict that dynamin loss of function mutation (*dyn-1(lf)*) or dynamin knockdown by RNAi (*dyn-1(RNAi)*) might confer on the worms an enhanced resistance to *P. aeruginosa*. However, this is not the case. The complicating factor is that the overall fitness of the *dyn-1(lf)* mutants is compromised, such that their survival is reduced on exposure to non-pathogenic *E. coli* HB101, as well as to *P. aeruginosa*. We interpret this loss of fitness to reflect the impairment of diverse cellular processes that depend upon dynamin-mediated endocytosis. Dynamin is essential for a broad array of endocytic events, including formation of synaptic vesicles in the nervous system (PMID: 9294229, 9802908), intestinal uptake of nutrients (PMID: 23799580) and phagocytosis (PMID: 16740477). We suggest that impairment of these processes by mutation or RNAi of *dyn-1* may offset any benefit to the worm of reduced H69 cleavage.

*Minor Comments:*

*1) The statement in line 47-48 is a bit misleading. A mutation in the toxA gene does appreciably alter virulence towards certain C. elegans mutant backgrounds (such as zip-2). Perhaps one can say specifically that it does not alter virulence for "wild type" or "N2."*

**Response**:

We have amended the sentence in question (lines 46 - 48), to clarify that it refers to the wild type background. The sentence now reads: “However, ToxA is likely not the only translational inhibitory activity in *P. aeruginosa* for *C. elegans*, since mutation of ToxA does not appreciably abrogate a host response to translation inhibition and does not alter virulence towards wildtype *C. elegans* [11]. ”

*2) Line 161: Should be Figure S2C.*

**Response**:

The error in line 161 has been corrected.

**Responses to Reviewer #2**

***Reviewer #2:***

*For this revised MS, I have not seen the reviewers' comments on the previous version, nor the authors' rebuttal.*

*This is a clear and logical examination of the potential for PA14 to provoke specific cleavage of 26S rRNA in C. elegans. It represents a real step forward in our understanding of a virulence strategy and how a surveillance-type immune response is triggered. Even in the absence of mechanistic details (both on the pathogen and host sides) it will be of relatively broad interest.*

**Response**:

We appreciate the reviewer’s acknowledgment of the manuscript as clear, logical and of broad interest. Our revisited manuscript and the responses below address the reviewer’s concerns.

*I only have very minor comments.*

*"Several lines of evidence support the idea that H69 rRNA cleavage is a component of P. aeruginosa virulence towards C. elegans…..Third, worms carrying mutations in the pmk-1, zip-2 and fshr-1 genes, which impair three distinct pathways required for resisting PA14 infection, show both reduced survival on PA14 [15,17,19] and enhanced levels of H69 cleavage (Fig 3A).*

*As shown by Miller et al. (10.1371/journal.pone.0137403; not cited), for fshr-1, that reduced survival on PA14 is correlated with an increased bacterial load in the gut. The same is true for pmk-1 and zip-2. The enhanced levels of H69 cleavage here could thus very simply be secondary consequence and this does not to my mind represent a line of evidence.*

**Response**:

The nature of the presented evidence is indeed correlative. However, the fact that genetic impairment of the three pathways mediated by *pmk-1*, *fshr-1* and *zip-2*, leads in all three cases to enhanced H69 cleavage, is consistent with H69 cleavage being a component of *P. aeruginosa* virulence.

The increased accumulation of bacteria in the gut of fshr-1, pmk-1 and zip-2 mutants compared to wildtype worms supports the idea that these three genes promote expression of host immune factors that restrict bacterial establishment or growth inside the nematode host. It can be envisioned that increased ribosomal cleavage results from the increased accumulation of P. aeruginosa in the gut. Yet, in this hypothetical scenario, H69 cleavage would still be a consequence of bacterial virulence.

We have now modified the manuscript to cite the work of Miller et al. that described the involvement of the *fshr-1* pathway as a host response to *P. aeruginosa* PA14.

*I take issue with the first sentence that starts, "Pathogens such as P. aeruginosa adventitiously modify animal host physiology". It is unlikely that these modifications are adventitious. They are more likely to be the result of a long process of selection.*

**Response**:

We agree with the reviewer’s view that bacterial-driven modifications of animal host physiology are often the result of long evolutionary processes. We had mistakenly used the word ‘adventitiously’ when we meant ‘advantageously’.

*including nematodes such as C. elegans and vertebrates*

*including vertebrates and nematodes such as C. elegans*

**Response**:

The sentence has been rephrased as suggested by the reviewer (lines 57-58)

**Responses to Reviewer #3**

***Reviewer #3:***

*Vasquez-Rifo et al. characterize a mechanism by which pathogenic Pseudomonas aeruginosa inhibits protein synthesis of the host C. elegans. The authors demonstrate using capillary electrophoresis that exposure to P. aeruginosa results in the accumulation of ribosomes that have undergone specific cleavage at helix 69 of the 26S ribosomal RNA, a component of the ribosome decoding center. H69 cleavage is shown to activate ZIP-2-dependent signaling. Prior studies have shown that translational inhibition activates ZIP-2-dependent signaling, and the authors' results confirm this to be the case during H69 cleavage by P. aeruginosa. The cleavage is also independent of the previously characterized P. aeruginosa toxin ToxA.*

*The quantitative capillary electrophoresis analysis impressively demonstrates specific cleavage of the 26S rRNA, and experiments demonstrate the dependence of this cleavage on the virulence of the Pseudomonas aeruginosa, and the enhancement of the cleavage in genetic backgrounds in which pathogen resistance is impaired. Cleavage is specific to P. aeruginosa and is not observed during infection by other pathogenic bacteria. Notably, the authors also demonstrate a dramatic reduction in total RNA content during P. aeruginosa infection.*

**Response**:

We are thankful for the reviewer’s critique and assessment of our findings. Our revisited manuscript and the responses below address the reviewer’s concerns.

*The bacterial or host enzyme catalyzing cleavage remains undefined. Interestingly, the cleavage event requires activity of DYN-1, suggestive of a dependence of cleavage on mechanisms of endocytic internalization. The results in the manuscript are of considerable interest, as one could envision a similar process occurring in cells of other host organisms under attack by P. aeruginosa, and bacterial virulence effector mechanisms continue to yield basic insights into both pathogen and host biology. The story would be greatly strengthened through identification of the bacterial factors involved. Many ribotoxic effectors produced by bacteria have been characterized, and one might anticipate either through sequence homology searching or direct analysis of the P. aeruginosa PA14 mutant library, further mechanistic insight might be derived. This is a lot to ask, but at the same time, simply the characterization of a cleavage event seems a bit preliminary, like the opening act of a*

*more complete story.*

**Response**:

We share the reviewer's interest in the identity of the nuclease involved in the rRNA cleavage at H69, and acknowledge that identification of the nuclease would enhance the manuscript's narrative. However, we think that none of the presented claims or conclusions in the manuscript critically depends on such identification.

Our work extends beyond the characterization of H69 cleavage, as we report mechanistic insights into the phenomenon (i.e. dependence on bacterial quorum sensing and other virulence regulators such as gacA and PA14_27700; as well as dependence on host dynamin function). Also, we experimentally support that activation of the host *zip-2* pathway in response to H69 cleavage, a biological consequence of ribosome cleavage at H69 and an endogenous readout of the induced translational inhibition.

We have already evaluated a multitude of bacterial factors using available mutants (i.e. PA14 transposon library, PMID: 16477005) and we report the effect on H69 cleavage for a biological selection of the mutants tested. The only bacterial factors that we have found to be required for H69 cleavage are gene regulators (i.e. lasR, rhlR, gacA, PA14_277700 shown in Figure 2). We also report tests of mutants that impair bacterial secretion systems and well-known *P. aeruginosa* toxins (Figure S4).

As mentioned by the reviewer, a multitude of toxic bacterial ribonucleases are known. However, many of these toxins are either not present in *P. aeruginosa* or are found at low frequencies among *P. aeruginosa* isolates. We have found that *P. aeruginosa* PA14, a strain that induces strong H69 cleavage, lacks homologs of the ribonucleases mazF, relE, higB, yoeB, pemK, colicin E3, pyocin S6 and barnase. We have tested available mutants of annotated PA14 nucleases and have as yet found no candidate gene for the H69 nuclease.

It is apparent to us that a very substantial amount of work is required to achieve conclusive identification of the nuclease directly responsible for H69 cleavage, and is beyond the scope of the current report.

**Responses to Reviewer #4**

***Reviewer #4:***

*In this manuscript, "Pseudomonas aeruginosa cleaves the decoding center of Caenorhabditis elegans ribosomes", the authors describe an extraordinary intriguing and exciting mechanism by which P. aeruginosa inhibits protein synthesis in its host C. elegans by cleaving the 26S rRNA specifically at helix 69. This cleavage of 26S rRNA at H69 results in the activation of the host defense response, through what appear to be novel mechanism(s). Although these latter data are more correlative, the authors convincingly show that H69 cleavage and host immune activation does not occur in a straightforward way through known mechanisms. Finally, the authors convincingly argue that the 26S rRNA cleavage is a mechanism by which the pathogen abrogates the host defense and antibacterial response. The manuscript describes novel, original and exciting biology. The experiments are well thought through and the results are convincing. The manuscript is very well written, and I personally*

*appreciated the attentiveness with which the authors discussed their results.*

**Response**:

We appreciate the reviewer’s opinion that our presented research is extraordinary and novel, our results are convincing, and the manuscript is well-written. Our revisited document and the responses below address the reviewer’s concerns.

*I have only one minor concern regarding experiments. The authors conduct experiments that suggest that the 26S rRNA cleavage occurs at intact ribosomes. The authors conclude this from experiments depicted in S1A Fig showing that an rRNA-free S100 extract prepared from P. aeruginosa infected animals can cleave ribosome-associated rRNA from unexposed animals. These experiments would be more convincing if the authors included data showing the profile of the rRNA-free S100 prepared from P. aeruginosa infected animals prior to incubation with intact ribosomes.*

**Response**: We have included the RNA profile of the S100 extract (prepared from PA14-exposed worms) that was used in the in vitro assays. The data has been added to the supplemental figure 2A.

*Minor points*

*1) Figure S1 is discussed in the manuscript after Figure S2.*

**Response**:

The supplemental figures S1 and S2 have been renamed to reflect the order of citation in the manuscript. All text references to the figures have been updated accordingly.

*2) Line 174: missing an 'of'.*

*3) Line 358: should be S100.*

**Response**:

The errors found in lines 174 and 358 have been fixed accordingly.
