# Supplementary material for: Pseudomonas aeruginosa cleaves the decoding center of Caenorhabditis elegans ribosomes
Source: PLoS Biol. 2020 Dec 1;18(12):e3000969. doi: 10.1371/journal.pbio.3000969 (PMC7707567; doi:10.1371/journal.pbio.3000969)
Supplement: Attachment — Submitted filename: Response_to_Reviewers_10_07_2020.docx [file pbio.3000969.s013.docx]

**Responses to reviewer report**

A response to the reviewers' comments is not applicable
